# Diversity, functionality, and stability: shaping ecosystem multifunctionality in the successional sequences of alpine meadows and alpine steppes on the Qinghai-Tibet Plateau

**DOI:** 10.3389/fpls.2025.1436439

**Published:** 2025-03-13

**Authors:** Xin Jin, Abby Deng, Yuejun Fan, Kun Ma, Yangan Zhao, Yingcheng Wang, Kaifu Zheng, Xueli Zhou, Guangxin Lu

**Affiliations:** ^1^ College of Agriculture and Animal Husbandry, Qinghai University, Xining, China; ^2^ Enterprise High School, Redding, CA, United States; ^3^ Qinghai Vocational and Technical Institute of Animal Husbandry, Xining, China; ^4^ Qinghai Province Grassland Station, Xining, China

**Keywords:** alpine meadow, diversity, functionality, stability, ecosystem multifunctionality

## Abstract

Recent investigations on the Tibetan Plateau have harnessed advancements in digital ground vegetation surveys, high temporal resolution remote sensing data, and sophisticated cloud computing technologies to delineate successional dynamics between alpine meadows and alpine steppes. However, these efforts have not thoroughly explored how different successional stages affect key ecological parameters, such as species and functional diversity, stability, and ecosystem multifunctionality, which are fundamental to ecosystem resilience and adaptability. Given this gap, we systematically investigate variations in vegetation diversity, functional diversity, and the often-overlooked dimension of community stability across the successional gradient from alpine meadows to alpine steppes. We further identify the primary environmental drivers of these changes and evaluate their collective impact on ecosystem multifunctionality. Our analysis reveals that, as vegetation communities progress from alpine meadows toward alpine steppes, multi-year average precipitation and temperature decline significantly, accompanied by reductions in soil nutrients. These environmental shifts led to decreased species diversity, driven by lower precipitation and reduced soil nitrate-nitrogen levels, as well as community differentiation influenced by declining soil pH and precipitation. Consequently, as species loss and community differentiation intensified, these changes diminished functional diversity and eroded community resilience and resistance, ultimately reducing grassland ecosystem multifunctionality. Using linear mixed-effects model and structural equation modeling, we found that functional diversity is the foremost determinant of ecosystem multifunctionality, followed by species diversity. Surprisingly, community stability also significantly influences ecosystem multifunctionality—a factor rarely highlighted in previous studies. These findings deepen our understanding of the interplay among diversity, functionality, stability, and ecosystem multifunctionality, and support the development of an integrated feedback model linking environmental drivers with ecological attributes in alpine grassland ecosystems.

## 1 Introduction

Global biodiversity is declining unprecedentedly due to climate change and human activities, significantly impacting ecosystem multifunctionality (EMF)—the capacity of ecosystems to provide multiple functions and services simultaneously (Midgley, 2012). EMF is influenced by climatic factors, species diversity, and functional diversity (Wolf et al., 2021). For instance, in dryland ecosystems, mean annual precipitation strongly affects EMF, whereas mean annual temperature has a weaker impact (Delgado-Baquerizo et al., 2016). Conversely, on the QTP, precipitation does not significantly influence EMF, while temperature has a pronounced positive effect (Wu et al., 2022). These differences highlight that EMF mechanisms vary across ecosystems due to distinct interactions between environmental factors and biotic communities. Additionally, species and functional diversity are closely linked to EMF. Environmental stress reduces species diversity and forces vegetation functional traits to converge, decreasing functional diversity and thereby impairing ecosystem functioning (Biswas and Mallik, 2011). In grassland communities, disturbances often lead to the loss of certain species, while others compensate by increasing their abundance or functional roles, enhancing community resilience and stability (Wu et al., 2022). Consequently, the interactions among climate, species diversity, functional diversity, stability, and EMF are highly complex and ecosystem-specific.

Approximately half of the world’s grassland ecosystems are currently undergoing degradation, with approximately five percent facing severe to extreme levels of deterioration (Wang et al., 2005). The Tibetan Plateau (QTP) is recognized as a crucial region for the conservation of high-altitude biodiversity, underscoring its global ecological significance (Chen et al., 2013; Myers et al., 2000). Within the QTP, alpine meadows are key contributors to species diversity (Wu et al., 2022); however, they exhibit relatively low stability, resistance, and resilience in the face of environmental stressors (Li et al., 2020; Yang et al., 2022). Extensive degradation driven by overgrazing and anthropogenic interference has compromised nearly one-third of the natural grassland area (Hou et al., 2022). This widespread deterioration not only threatens the unique biodiversity of the QTP but also undermines the essential ecosystem services provided by these high-altitude grasslands. Addressing these challenges is imperative for the sustainable conservation and restoration of grassland ecosystems on the Tibetan Plateau.

The complex topography and high spatial heterogeneity of the QTP, coupled with limited study durations, have constrained vegetation studies to small-area ground surveys. Previous research has primarily focused on degradation gradients at the spatial scale while neglecting the temporal dimensions of degradation succession (Liu et al., 2017). There is a prevalent view that alpine vegetation on the QTP tends to evolve towards a drier final ecosystem, transitioning from alpine swamp meadow through meadow, steppe, desert steppe, to desert (Qin and Ding, 2009). Vegetation on the Qinghai-Tibet Plateau was reported to be highly dependent on specific climatic conditions and extremely sensitive to climate change (Zhang et al., 1996). Correspondingly, local field studies have observed a shift from alpine steppe to alpine meadow under warmer and wetter conditions, driven by enhanced hydrothermal dynamics (Wang et al., 2022). Conversely, with decreases in precipitation and temperature, the plant community structure of alpine meadows becomes simplified, leading to a transition from alpine meadows to alpine steppes (Zhao et al., 2011; Wang et al., 2022b; Zong et al., 2019). Recent studies using multivariate data fusion and deep learning have identified a 40-year successional trend between alpine meadows and steppes. Under annual precipitation ≤400 mm, steppes were dominant from 1979 to 1990, shifting to meadows dominance from 2010 to 2018 (Wang et al., 2023b). Climate change primarily drives the conversion between adjacent successional stages of alpine meadows and steppes. However, these ecosystems are often studied independently, overlooking their successional relationship (Shuren et al., 2022; Wang et al., 2023). This approach limits our understanding of vegetation and community dynamics in alpine grasslands. Therefore, examining species and community differences and their driving factors within this successional sequence is essential for enhancing our theoretical understanding of changes in alpine meadows.

We hypothesize that the succession from alpine meadows to steppes results in declines in species diversity, functional diversity, and community stability, ultimately reducing EMF. We propose that vegetational succession alters habitat conditions by decreasing precipitation or increasing temperatures, which, in turn, reduces soil nutrient availability. These environmental changes are expected to decrease vegetation diversity and restructure community composition, thereby diminishing functional diversity and stability. Ultimately, these ecological shifts are anticipated to impair EMF. This conceptual framework aims to clarify the complex responses of EMF to vegetation succession under changing climatic conditions on the Tibetan Plateau.

In this investigation, we examine alpine meadows and steppes in the Qilian Mountains of the QTP as a sequence of ecological degradation. Utilizing field surveys, we explore the interactions among climate factors, biodiversity, functional diversity, community stability, and EMF within these high-altitude ecosystems. Our objectives are to: 1) Characterize variations in habitat conditions, species diversity, community β-diversity, functional diversity, community stability, and EMF along the degradation gradient. 2) Identify the primary drivers of changes in species diversity, community β-diversity, functional diversity, and stability. 3) Elucidate the relationships between EMF and species diversity, community β-diversity, functional diversity, and community stability, thereby uncovering the key factors and regulatory pathways that influence EMF.

## 2 Materials and methods

### 2.1 Study area

The study was conducted in the Qilian Mountains, a key region of the QTP, where degraded grasslands comprise 72.4% of the total grassland area, indicating severe degradation (Luo et al., 2018). Previous research has utilized spatial sequences rather than temporal ones to investigate the succession from alpine swamp to meadows and ultimately to alpine steppe (Wu et al., 2022). This spatial approach facilitates long-term monitoring by comparing sites at different successional stages to infer ecological changes across degradation levels. Accordingly, we employed a spatial sequence method to examine the adjacent successional stages of Alpine meadows (AM) and alpine steppes (AS). Fieldwork was conducted during the peak biomass period from late July to early August 2021 across the Qilian Mountains region (97.252°E to 102.592°E, 36.693°N to 37.453°N), spanning 482.23 km (**Supplementary Figure 1**). The study area ranges in elevation from 2,510 to 4,045 meters, with average annual temperatures between -7.50°C and -0.39°C and annual precipitation from 236.18 mm to 550.34 mm (**Supplementary Table 1**).

### 2.2 Plant community survey and trait measurement

The sampling design was based on the BIODESERT survey method (Maestre, 2017) and adapted to the QTP’s complex topography. To ensure representativeness, we maximized the number of alpine meadow plots to comprehensively capture alpine environmental characteristics. Alpine steppe plots were subsequently selected to retain key species from meadow communities, reflecting their successional sequence. Vegetation surveys were conducted during the peak biomass period (late July to early August 2021) across 32 sites, including 23 alpine meadows and 9 alpine steppes, each separated by at least 4.90 km. Within each site, a randomly designated 30 m × 30 m plot contained fifteen randomly placed 0.5 m × 0.5 m ecological quadrats. In each quadrat, we recorded species presence, abundance, cover (using the pin-prick method), and the average height of ten specimens. Unidentified plants were photographed in the field and later identified using the Chinese Plant Image Library (https://ppbc.iplant.cn/) and Flora of China (https://www.iplant.cn/frps). Subsequently, we collected all aboveground and belowground biomass from each quadrat. After oven-drying both components to a constant weight, we combined them to calculate the total biomass.

In each ecological plot, six well-developed specimens of each species were selected for trait measurements, following methodologies from previous grassland studies assessing functional traits across degradation gradients (Saruul et al., 2019). We identified ten key plant traits aligned with our study objectives, categorized under two resilience mechanisms: community resilience (ET) and community resistance (RT). Community resilience traits include lifespan, life form, flowering duration, leaf area, leaf dry matter content, and leaf phosphorus content. Community resistance traits encompass lifespan, leaf hair type, plant height, leaf dry matter content, leaf phosphorus content, leaf carbon-to-nitrogen ratio, and leaf lignin content. Notably, lifespan, leaf dry matter content, and leaf phosphorus content were highlighted as critical indicators of community resilience and stability (Saruul et al., 2019). Plant height and leaf area were measured in the field using a portable laser leaf area meter (LI-3000C, LI-COR). To ensure data accuracy, leaf phosphorus content, leaf carbon-to-nitrogen ratio, leaf dry matter content, and leaf lignin content were analyzed in the laboratory following the methods described by Saruul et al. (2019).

### 2.3 Soil characterization measurement

Following the vegetation survey, soil samples were collected, as primary physiological and biochemical activities in alpine grasslands occur mainly in the surface soil (Liu et al., 2018). Soil bulk density was measured using a 100 cm³ ring knife on samples from the 0–15 cm layer. For soil physicochemical analysis, samples from the same layer were obtained with a 7 cm diameter auger, combining five samples per quadrat into a composite sample. The measured parameters included bulk density (BD), ammonium nitrogen (NH_4_^+^-N), nitrate nitrogen (NO_3_^-^-N), total nitrogen (N), organic matter (OM), available phosphorus (AP), available potassium (AK), and soil pH (Tonin et al., 2019). Additionally, soil moisture (SM), soil temperature (ST), and soil electrical conductivity (EC) were measured in the field using a TDR 350 soil moisture meter, with five measurements per quadrat at a depth of 0–15 cm to match the soil sampling depth.

### 2.4 Geography, climate, topography and grazing

To address spatial autocorrelation and effectively analyze the geographic structure of the sampling sites, latitude and longitude were decomposed into Moran eigenvectors (MEMs) using the dbmem function in the R software package “spatial” (Dray et al., 2017). A spatial vector representing broader geographic features (MEM3 was calculated, *P* < 0.01) was selected for data analysis.

Mean temperature and precipitation in Qilian Mountain National Park (1961–2020) have shown significant increasing trends (*P* < 0.05) (Wang et al., 2022a). Consequently, we used average annual temperature (MAT) and precipitation (MAP) to characterize the area’s climate. Following the methodology of Saruul et al. (2019), we recorded the geographic coordinates of each sampling site using GPS. Climate factors were extracted for each site using ArcGIS 10.8 and climate raster data (2000–2021) with a 1 km spatial resolution, which is finer than the 4.90 km minimum distance between sampling points, ensuring data accuracy. Climate raster data were sourced from the National Earth System Science Data Sharing Service Platform (http://loess.geodata.cn).

The altitude of each sampling point was measured using the Global Positioning System (GPS). A 90-meter resolution Digital Elevation Model (DEM) was employed for the study area to ensure data accuracy, considering the minimum distance of 4.90 km between sampling sites. Using ArcGIS 10.8, two key topographic variables, aspect and slope, were extracted from the DEM (Saruul et al., 2019. The DEM was obtained from the National Glacial Tundra Desert Science Data Centre (http://www.ncdc.ac.cn).

Grazing pressure in the area was quantified as sheep units per unit area (Saruul et al., 2019). Using ArcGIS 10.8, cattle and sheep density data, as well as cool- and warm-season pastures, were extracted from livestock raster data based on the coordinates of the sampled area. Field observations were combined with this data to determine whether the sampling area corresponded to cool- or warm-season pastures. The livestock raster data, which cover rangeland extent and sheep and cattle populations on the Tibetan Plateau in 2020, were released in 2023 by the Second Tibetan Plateau Scientific Expedition and Research Program (TPSERP) and have a spatial resolution of 500 meters. This resolution is sufficient to meet the minimum 4.90 km distance between sampling sites, thereby ensuring data accuracy. Livestock density data were converted into standard sheep units (1 cattle = 5 sheep) to quantify grazing pressure as the number of sheep per unit area. The data are available as Geo TIFF files on the Zenodo platform: https://doi.org/10.5281/zenodo.7692064.

### 2.5 Diversity index, functional diversity and stability of plant communities

Species diversity was assessed using the Patrick index, Shannon–Wiener index, and Pielou index, with species importance values calculated based on their relative height, cover, and biomass (Zhang et al., 2018b).

The functional diversity of grasslands, determined by the abundance and functional traits of vegetation species, can be evaluated using key indicators such as Functional Richness (FRic) (Cornwell et al., 2006), Functional Evenness (FEve) (Mouillot et al., 2005), Functional Divergence (FDiv) (Mouillot et al., 2005), and Functional Dispersion (FDis) (Laliberté, and Legendre, 2010). These metrics are calculated using the FD package (Laliberté, and Legendre, 2010) within the R software.

Vegetation community stability was assessed using indicators of community resistance (RT), community resilience (ET), structural variability (St), and functional variability (Fu). These classifications were derived from 10 functional traits identified in our quadrat surveys, following approaches established in grassland studies (Cornelissen et al., 2003; Laliberté, and Legendre, 2010; Sterk et al., 2013). Among these traits, ET is determined by factors such as life cycle, life form, flowering duration, leaf area, and phosphorus content, while RT is influenced by traits including leaf hair type, plant height, carbon-to-nitrogen ratio, and lignin content. Data for life cycle, life form, leaf hair type, and flowering time were gathered through field observations and cross-referenced with the Flora of China (http://www.efloras.org/), with trait values assigned as per Saruul et al. (2019). Structural variability (St) was quantified using the mean Jaccard dissimilarity of species presence/absence, capturing the degree of variation in community composition (Pestana et al., 2019). Functional variability (Fu) was determined based on the spatial mean and standard deviation of plant biomass at each site (Tilman et al., 2006). The specific formulas for calculating RT, ET, St, and Fu were referenced from studies examining grassland degradation gradients (Bai et al., 2022).

### 2.6 Ecosystem multifunctionality of grassland

Traditional research often emphasizes single-scale indicators, capturing certain aspects of multifunctionality but failing to account for the interconnectedness of ecosystems as complex, multifaceted systems (Siwicka et al., 2021). This study investigates the effects of alpine meadows and alpine steppes, considered as contiguous successional sequences, on vegetation-related EMF. The analysis incorporates multiple factors, including vegetation biomass (both aboveground and belowground), plant diversity (encompassing species diversity (α-diversity), community diversity (β-diversity), and functional diversity), as well as community stability indicators. To address the issue of non-normal data distribution, all indicators were normalized to improve the accuracy and reliability of statistical analyses (Maestre et al., 2012), and Z-scores were subsequently calculated. EMF was quantified using the mean value method (Valencia et al., 2015).

### 2.7 Statistical analyses

Prior to performing two-sample t-tests to compare environmental parameters, productivity, species diversity, functional diversity, stability, and EMF, we first checked the data for normality and homogeneity of variances to ensure that the underlying t-test assumptions were met. Spearman correlations among species diversity, functional diversity, stability, and EMF were computed using the ‘Hmisc’ package, and chord diagrams produced with the ‘circlize’ package illustrated their interrelationships. Subsequently, vegetation communities were analyzed using principal coordinate analysis (PCoA) and biaxial non-metric multidimensional scaling (NMDS), both based on species’ relative abundance and Bray–Curtis distances, implemented through the ‘vegan’ package (Oksanen, 2010). In addition, community dissimilarity, also derived from Bray–Curtis distances, was evaluated using permutation multivariate analysis of variance (PERMANOVA) and analysis of similarity (ANOSIM).

To determine key environmental factors influencing α-diversity (Patrick index) and β-diversity (Bray–Curtis dissimilarity) during succession (Lazzaro et al., 2020), we employed a linear mixed-effects model, in which vegetation type, climate, grazing, spatial, geographic, and soil parameters were included as fixed effects, and plot repetition was treated as a random effect. Variance inflation factors (VIF) were applied to address collinearity, excluding any predictor with a VIF greater than 10. The optimal model was selected via complete subset regression based on corrected Akaike Information Criterion (AICc) values using the MuMIn package (García-Palacios et al., 2018). Finally, hierarchical partitioning with the glmm.hp package was employed to quantify each predictor’s relative contribution to explained variance, expressed as a percentage of the _Con_R² (Conditional R²) (Lai et al., 2023).

To clarify the key effects of environmental factors on variations in functional diversity and community stability, we performed Spearman correlation analyses between environmental factors and both functional diversity and community stability, presenting the results using heatmaps. The overall influence of environmental factors on functional and stability indices was assessed through multiple linear regression using the lm function and illustrated in bar charts. The significance of individual environmental factors was determined through variance decomposition analysis in conjunction with multiple linear regression models, computed with the calc.relimp function from the relaimpo package (Jiao et al., 2020), and depicted by the size of circles.

To prevent any single predictor (e.g., habitat, vegetation diversity, functional diversity, stability) from being overshadowed when identifying the drivers of EMF, each factor was analyzed separately (Manning et al., 2018). First, a random forest model, implemented with the rfPermute function, was used to pinpoint significant predictors of EMF, with their significance assessed through 999 random permutations. Subsequently, model importance was evaluated using the A3 package (Jiao et al., 2018). Building on these identified predictors, a linear mixed-effects model (in which vegetation type was included as a fixed effect, and plot repetition was treated as a random effect) was then developed to examine the relative influence of habitat, vegetation diversity, functional diversity, and stability on changes in EMF. To further clarify the contributions of each predictor, hierarchical partitioning in the glmm.hp package was applied, thereby quantifying each factor’s contribution to the _Con_R² (Conditional R²) (Lai et al., 2023).

Finally, to understand the complex interplay among these factors, a mixed-effects piecewise structural equation model (SEM), treating different sampling points as random effects, was constructed. This allowed us to assess how habitat, vegetation diversity, functional diversity, stability, and their interactions jointly influence EMF. All statistical analyses and figure generation were performed using R 4.2.3 (R Development Core Team, 2023).

## 3 Results

### 3.1 Alterations in habitat

We identified 17 environmental variables representing climate, grazing, geography, and soil physicochemical properties as proxies for grassland habitats. These environmental indicators have changed throughout the ecological succession between alpine meadows (AM) and alpine steppes (AS). Specifically, the AM had a significantly higher MAP than the AS, with lower MAT, pH and altitude (*P* < 0.05). Moreover, there were increases in EC from AM to AS, while grazing, slope, NH_4_^+^-N, NO_3_^–^N, N, OM, AP, AK, BD, ST, and SM showed decreasing trends (**Table 1**).

**Table 1:** The alteration of habitat factors in alpine meadows and alpine steppe.

Environmental factors	Variables	Alpine meadow (AM)	Alpine steppe (AS)
**Climate**	MAP (mm)	501.52 ± 36.65^a^	355.51 ± 19.90^b^
	MAT (°C)	-4.44 ± 2.12^b^	-3.37 ± 2.17^a^
**Grazing**	Grazing(sheep/ha)	18.24 ± 3.23^a^	5.76 ± 0.58^b^
**Geography**	Aspect (°)	167.31 ± 57.79^a^	217.55 ± 74.87^a^
	Slope (°)	9.19 ± 7.30^a^	4.67 ± 3.44^a^
	Altitude (m)	3381.24 ± 351.19^b^	3600 ± 106.49^a^
**Soil physical-chemical factors**	NH_4_^+^-N (mg/kg)	106.42 ± 43.21^a^	81.96 ± 22.21^a^
	NO_3_-N (mg/kg)	86.53 ± 36.28^a^	83.40 ± 33.53^a^
	N (mg/kg)	6270.12 ± 2098.14^a^	5672.97 ± 2443.17^a^
	OM (%)	15.11 ± 7.82^a^	13.52 ± 8.70^a^
	AP (mg/kg)	31.45 ± 21.48^a^	25.96 ± 10.44^a^
	AK (mg/kg)	405.03 ± 176.58^a^	327.41 ± 85.88^a^
	pH	6.98 ± 0.58^b^	7.59 ± 0.39^a^
	BD (g/100cm^3^)	0.83 ± 0.25^a^	0.80 ± 0.27^a^
	ST (°C)	21.63 ± 4.59^a^	21.50 ± 3.49^a^
	SM (%)	38.34 ± 13.41^a^	36.18 ± 12.58^a^
	EC (μS/cm)	0.43 ± 0.26^b^	0.52 ± 0.29^a^

The values in table represent mean ± SE. Comparisons were made between two treatments, AM (n = 69) and AS (n = 27), using an independent two-sample t-test (df = 94). MAP, Mean annual precipitation; MAT, Mean annual temperature; Grazing, Grazing; Aspect, Aspect; Slope, Slope; Altitude, Altitude; NH_4_^+^-N, Soil ammonium nitrogen; NO_3_-N, Soil nitrate nitrogen; N, Soil total nitrogen; OM, Soil organic matter; AP, Soil available phosphorus; AK, Soil available kalium; pH, Soil pH; BD, Soil bulk density; ST, Soil temperature; SM, Soil moisture; EC, soil electrical conductivity. Different letters in the same row indicate that the variables differ significantly between grassland types, *P <*0.05.

Red indicates Climate factors, green indicates Grazing factors, blue indicates Geography factors, and peach indicates Soil physical-chemical factors.

### 3.2 Alterations and drivers of α diversity and β diversity in vegetation

The total biomass of vegetation was significantly higher in alpine meadows (AM) compared to alpine steppes (AS) (*P*<0.001, **Supplementary Figure 2A**). Additionally, the Patrick index, Pielou index and Shannon index for plants in AM were notably higher than those in AS (*P*<0.001, **Supplementary Figures 2B, C**; *P*<0.01, **Supplementary Figure 2D**). To examine changes in β diversity of plant communities between AM and AS, a Principal Coordinates Analysis (PCoA) based on Bray-Curtis distances was performed, revealing significant differences between AM and AS (PERMANOVA, *P*<0.01, **Supplementary Figure 2E**). Similarly, the Non-metric Multidimensional Scaling (NMDS) analysis (ANOSIM, *P*<0.01, **Supplementary Figure 2F**) supported the findings of the PCoA, showing notable changes in the plant communities during the succession.

**Figure 1:**
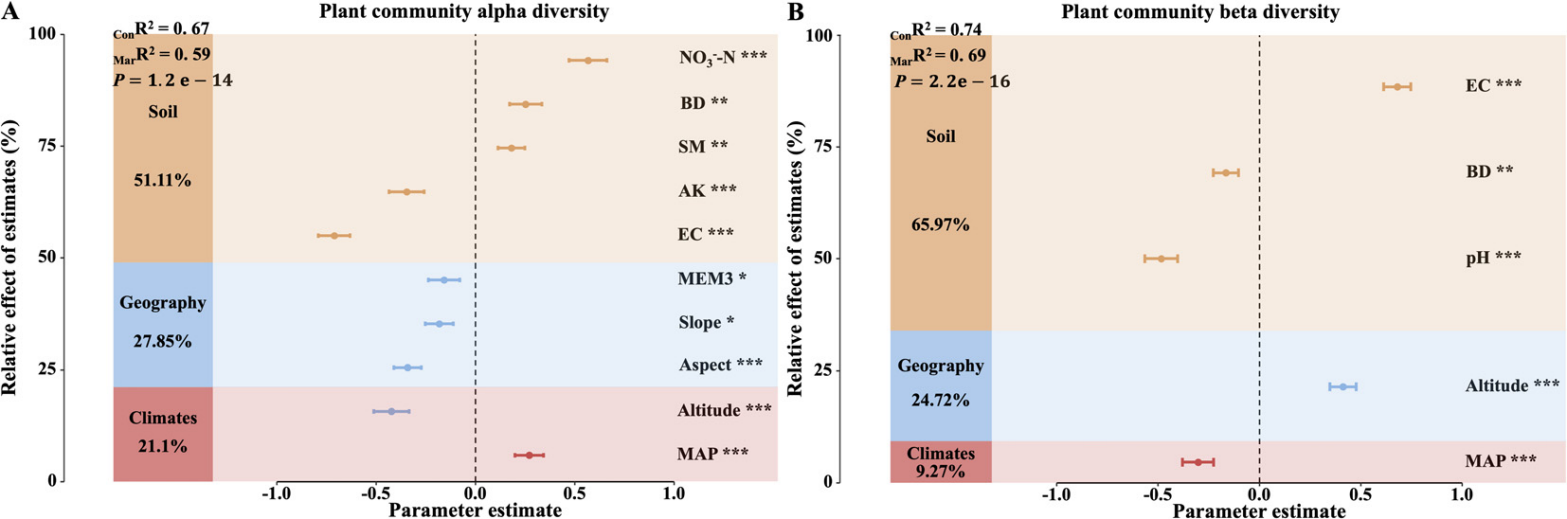
Drivers of differences in α-diversity and β-diversity between alpine meadows (AM) and alpine steppes (AS). **(A)** Relative contributions of habitat key factors to changes in α-diversity. **(B)** Relative contributions of habitat key factors to changes in β-diversity. _Mar_R² (Marginal R²) represents the proportion of variance explained by fixed effects alone, whereas _Con_R² (Conditional R²) denotes the variance explained by both fixed and random effects. The mean parameter estimates (standardized regression coefficients) of the predictors and their associated 95% confidence intervals, the relative importance of each predictor. MAP, Mean annual precipitation; Aspect, Aspect; Slope, Slope; Altitude, Altitude; MEM3, Spatial vector represents geography; NO_3_^–^N, Soil nitrate nitrogen; AK, Soil available kalium; pH, Soil pH; BD, Soil bulk density; SM, Soil moisture; EC, Soil electrical conductivity. Asterisks indicate the level of significance (*P < 0.05;**P < 0.01;***P < 0.001).

In the study of vegetation diversity changes during the degradation succession between AM and AS, we found that key factors explained 67% of the variance in α-diversity (_Con_R² = 0.67, *P*<0.001; **Figure 1A**), with soil factors contributing 51.11%, geographic factors 27.85%, and climatic factors 21.1%. Specifically, soil moisture (SM) and bulk density (BD) positively influenced α-diversity, while mean annual precipitation (MAP) and nitrate nitrogen (NO_3_^-^-N) had significant positive effects. In contrast, altitude, slope, aspect, soil exchangeable potassium (AK), and soil electrical conductivity (EC) had significant negative impacts, with slope and the third principal component of the Moran’s eigenvector map (MEM3) also exerting negative effects on α-diversity. Furthermore, climatic, geographic, and soil factors jointly explained 74% of the variation in vegetation β-diversity (_Con_R² = 0.74, *P*<0.001; **Figure 1B**), with soil factors being the most influential (65.97%), followed by geographic factors (24.72%) and climatic factors (9.72%). Notably, altitude and EC significantly increased β-diversity, while MAP, pH, and BD had significant negative effects.

### 3.3 Alterations and drivers of functional diversity and stability in vegetation

The functional dispersion index (Fdis), functional divergence index (Fdiv), functional richness (Fric), and functional evenness (Feve) were significantly higher in AM than in AS (*P* < 0.001; **Supplementary Figures 3A–D**). Similarly, the resilience (ET) and resistance (RT) of the community were significantly greater in AM compared to AS (*P* < 0.05; **Supplementary Figures 3E, F**). In contrast, both structural variability (St) and functional variability (Fu) were higher in AS than in AM, although these differences were not statistically significant (**Supplementary Figures 3G, H**).

**Figure 2:**
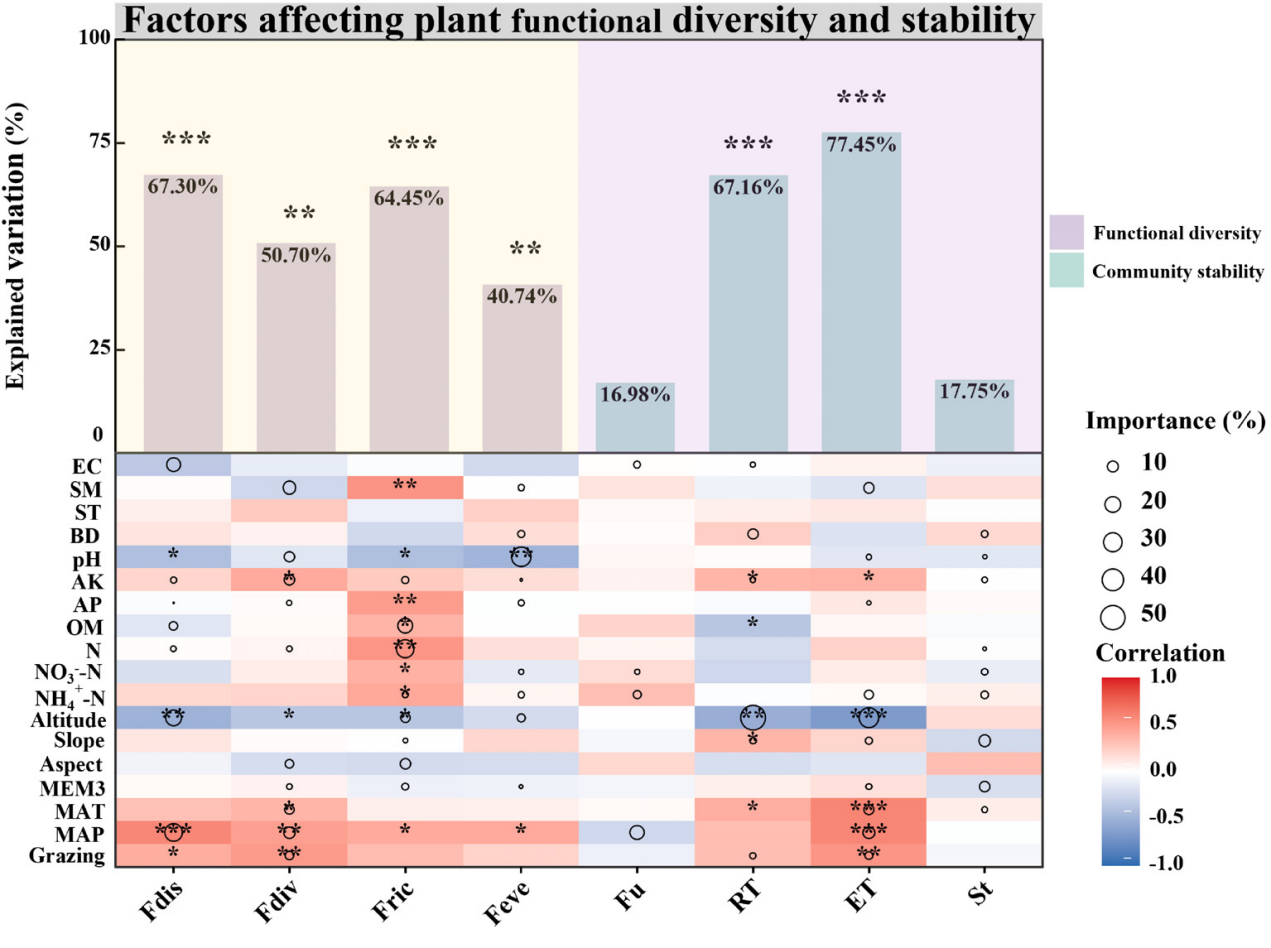
Drivers of differences in functional diversity and community stability between alpine meadows (AM) and alpine steppes (AS). Drivers of differences in functional diversity and community stability between alpine meadows (AM) and alpine steppes (AS). The bar chart represents the overall contributions of environmental factors, including specific contribution rates (%) and significance levels. The relative importance of each environmental factor is illustrated by the size of the circles in the diagram. Asterisks denote levels of statistical significance (*P < 0.05; **P < 0.01; ***P < 0.001). MAP, Mean annual precipitation; MAT, Mean annual temperature; Grazing, Grazing; MEM3, Spatial vector represents geography; Aspect, Aspect; Slope, Slope; Altitude, Altitude; NH_4_^+^-N, Soil ammonium nitrogen; NO_3_^–^N, Soil nitrate nitrogen; N, Soil total nitrogen; OM, Soil organic matter; AP, Soil available phosphorus; AK, Soil available kalium; pH, Soil pH; BD, Soil bulk density; ST, Soil temperature; SM, Soil moisture; EC, Soil electrical conductivity.

During the degradation succession, habitat factors significantly influenced the variability of vegetation community functional diversity indices (**Figure 2**). Specifically, habitat factors significantly affected the Fdis (*P* < 0.001), accounting for 67.30% of the variance, with dominant influences including MAP, Altitude, N, OM, AK and EC. Fdiv also showed significant habitat related variability (*P* < 0.01), explaining 50.70% of the variance with critical factors such as grazing, MAP, MAT, MEM3, Aspect, N, AP and AK. Fric was significantly shaped by habitat factors (*P* < 0.001), which explained 64.45% of the variation, primarily driven by MEM3, Aspect, Slope, Altitude, NH_4_^+^-N, N, OM and AK. Feve was significantly influenced by habitat (*P* < 0.01), which explained 40.74% of the variance. Altitude, NO_3_^-^-N, pH, and SM had negative effects, while NH_4_^+^-N and BD exerted positive influences (**Figure 2**).

The variability in community resistance (RT) was significantly shaped by habitat factors (*P* < 0.001; **Figure 2**), accounting for 67.16% of the variance, where altitude negatively influenced it, while slope, AK, BD and SE had positive influences. Habitat significantly determined the variation in resilience (ET) (*P* < 0.001; **Figure 2**), explaining 77.45% of the variance, with positive influences from Grazing, MAP, MAT, MEM3 and slope, while negative impacts were associated with pH and altitude. Habitat factors exerted a minimal effect on the variability of community structure (St) and functional variability (Fu), explaining merely 17.75% and 16.98% of the variance, respectively (**Figure 2**).

### 3.4 Correlations and contributions of habitat, diversity, functionality, and stability to ecosystem multifunctionality

The analysis of EMF within grassland ecosystems revealed that AM exhibited significantly higher multifunctionality than AS (*P* < 0.001; **Supplementary Figure 4A**). As AM and AS represent adjacent stages along a degradation successional sequence, vegetation α-diversity was positively correlated with Fdis, Fric, Feve, and RT (*P* < 0.05; **Supplementary Figure 4B**). In contrast, β-diversity was negatively correlated with Fdis, Fdiv, ET, RT, and EMF (*P* < 0.05; **Supplementary Figure 4B**). EMF was positively correlated with α-diversity, Fdis, Fdiv, Fric, Feve, ET, and RT (*P* < 0.05; **Supplementary Figure 4B**). Additionally, Fdiv was positively related to both Feve and ET, while Feve and ET were also positively correlated (*P* < 0.05; **Supplementary Figure 4B**). Furthermore, ET showed a positive correlation with RT (*P* < 0.05; **Supplementary Figure 4B**), whereas RT was negatively correlated with Fu (*P* < 0.05; **Supplementary Figure 4B**).

**Figure 3:**
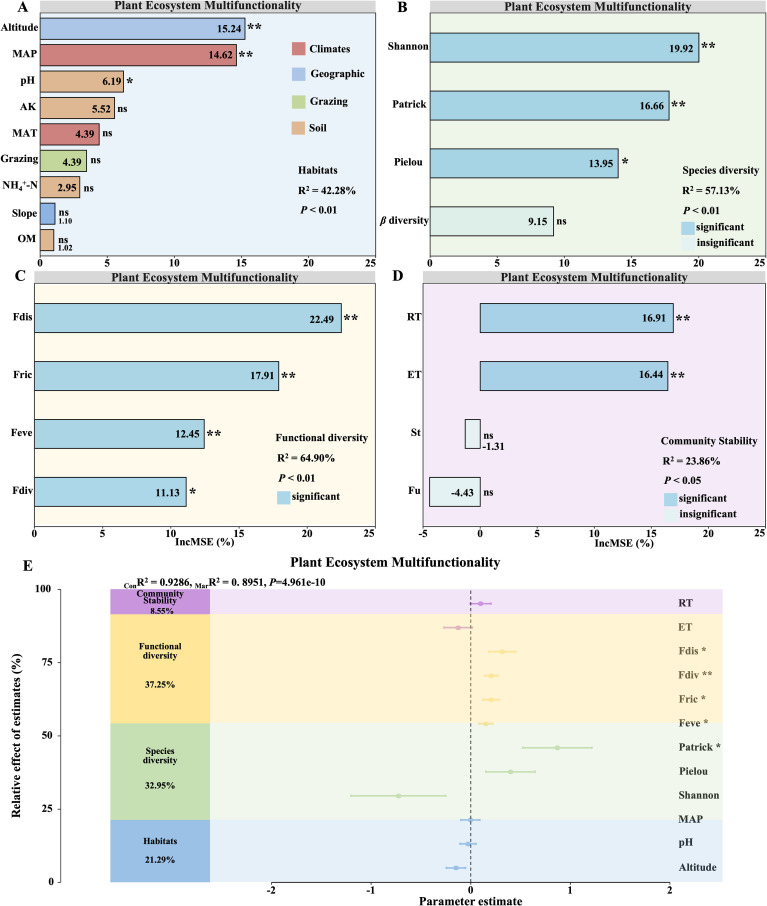
Determinants and contributions of key factors to grassland ecosystem multifunctionality. Random forest analysis reveals the major factors influencing ecosystem multifunctionality: Habitat **(A)**, Species diversity **(B)**, Functional diversity **(C)**, and Community Stability **(D)**. Panel **(E)** assesses key factors’ relative contributions to changes in ecosystem multifunctionality (EMF). It includes R^2^ values indicating model fit, mean parameter estimates with 95% confidence intervals, and the relative importance of predictors. _Mar_R² (Marginal R²) represents the proportion of variance explained by fixed effects alone, whereas _Con_R² (Conditional R²) denotes the variance explained by both fixed and random effects. Significance levels are denoted by asterisks (*P < 0.05; **P < 0.01).

Noteworthy contributors to EMF in alpine grassland ecosystems from habitat characteristics included MAP, altitude and pH (R^2^ = 42.28%; *P* < 0.01; **Figure 3A**). Among diversity metrics, primary influences were observed from the Shannon, Patrick and Pielou indices (R^2^ = 57.13%; *P* < 0.01; **Figure 3B**). Functional diversity indices (Fdis, Fdiv, Fric and Feve) were vital factors (R^2^ = 64.90%; *P* < 0.01; **Figure 3C**). Regarding stability characteristics, major contributors included RT and ET (R^2^ = 23.86%; *P* < 0.05; **Figure 3D**). This study further investigated the relative contributions of species diversity, functional diversity, and community stability to the variation in EMF (**Figure 3E**). The analysis revealed that habitat, species diversity, functional diversity, and community stability collectively explained 92.86%(_Con_R²) of the variance in EMF. Specifically, habitat factors accounted for 21.29% of the variance, species diversity for 32.95%, functional diversity for 37.25%, and community stability for 8.55% (**Figure 3E**). Notably, Fdis, Fdiv, Fric, Feve, and the Patrick index exhibited significant linear relationships with EMF, whereas other indicators showed no such associations. These findings underscore the complex interplay of factors influencing EMF, suggesting that while individual factors may have limited effects, the combined contributions of key composite factors are substantial.

**Figure 4:**
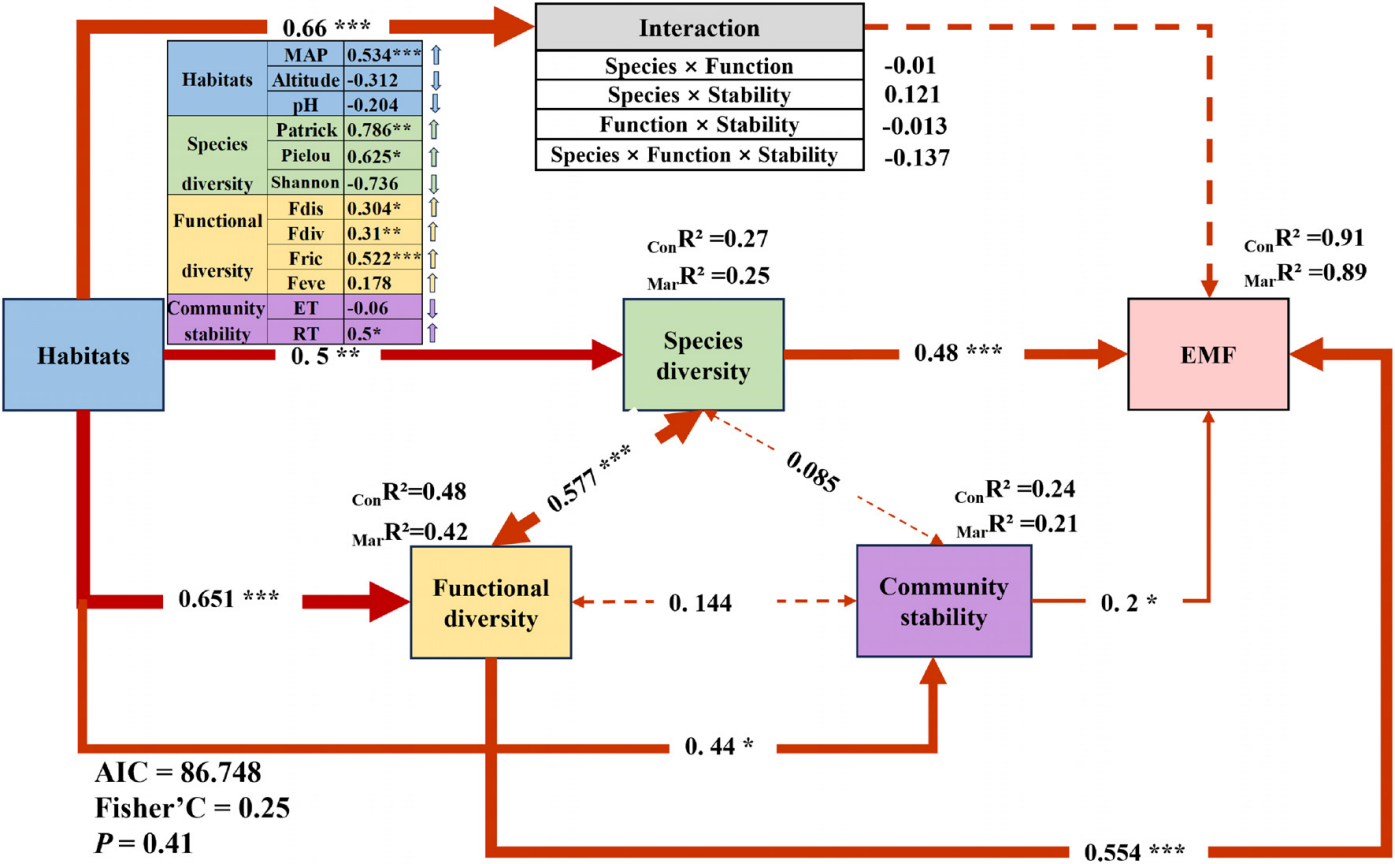
Ecosystem multifunctionality (EMF) and regulatory pathways in alpine grasslands. Structural equation modeling (SEM) reveals the direct and indirect effects of habitat, species diversity, functional diversity, and community stability on EMF. Path coefficients represent standardized effect sizes of these relationships. Red lines indicate positive effects, with line thickness reflecting the strength of the effect. Solid lines denote significant correlations, while dashed lines indicate non-significant correlations. _Mar_R² (Marginal R²) represents the proportion of variance explained by fixed effects alone, whereas _Con_R² (Conditional R²) denotes the variance explained by both fixed and random effects, with the random effects accounting for sampling points. Significance levels are denoted by asterisks: *P < 0.05, **P < 0.01, ***P < 0.001.

A structural equation model was developed using composite variables derived from the critical factors identified through random forest analysis. The results indicated that within habitat, increased precipitation, decreased altitude, and decreased pH exerted significantly positive effects on species diversity, functional diversity, community stability, and their interactions. Specifically, the habitat composite factor had the strongest effect on the interaction among species diversity, functional diversity, and community stability (0.66***), followed by functional diversity (0.651***), species diversity (0.5**), and community stability (0.44*) (**Figure 4**). In addition, the relationship between species diversity and functional diversity was the most pronounced (0.577***), followed by the interaction between functional diversity and community stability (0.144). The weakest correlation was between species diversity and community stability (0.085) (**Figure 4**). Notably, the interaction among species diversity, functional diversity, and community stability showed no significant linear effect on EMF. Among the composite factors, functional diversity exhibited the greatest influence on EMF (0.554***), followed by species diversity (0.48***), while community stability had a relatively weaker effect (0.2*) (**Figure 4**).

## 4 Discussion

### 4.1 Differences among habitat, diversity, functionality, stability, and ecosystem multifunctionality

The study examined alpine meadows and alpine steppes as contiguous successional sequences, revealing that higher temperatures and lower rainfall resulted in a warmer and drier climate transitioning from alpine meadows (AM) to alpine steppes (AS). Additionally, this climatic shift was accompanied by a decline in soil nutrients and a rise in altitude, leading to a decrease in vegetation α-diversity and a notable increase in β-diversity differences, ultimately decreasing ecosystem productivity significantly. Furthermore, the observations in this study align with other research, notably the transition from alpine marsh meadows to alpine meadows and then to alpine steppes, where decreases in precipitation and soil nutrients led to diminished productivity (Wang et al., 2023a). Functional diversity assesses how species within an ecosystem can substitute for or complement each other functionally, evaluating ecosystems’ overall functional performance based on their traits (Díaz and Cabido, 2001). Research has suggested that plant functional traits may indicate ecosystem stability (Liu et al., 2021).

Our research findings revealed that as AM transitioned to AS, there was an apparent decrease in functional diversity indices (Fdis, Fdiv, Fric, Feve), RT and ET, accompanied by increased community St and Fu variability. This change may be attributed to environmental selection favoring species and functional traits adaptable to a warmer, drier climate, accompanied by a marked decline in species and functional diversity, leading to a homogenization of functional traits. Although there were simultaneous increases in the stability (St) and functionality (Fu) variability of AS, these did not fully compensate for the loss of diversity, ultimately resulting in reduced resilience (RT) and ecosystem functionality (ET) of the community (Moorselvan Moorsel et al., 2021). Although RT remains consistent across degradation gradients within the same type of grassland (Bai et al., 2022), we observed a significant decrease in RT during the succession between AM and AS. We hypothesize that different grass types exhibit distinct RT characteristics. While RT may fluctuate within a range during the degradation of a single grass type, transitioning between grassland types (between AM and AS) is associated with a significant decline in RT. Functional traits are a crucial link between plants and their environment, impacting ecosystem functions (Fry et al., 2018). Our study revealed a significant decrease in EMF as degradation succession progresses, consistent with the substantial declines in species and functional diversity observed in this research. This supports the notion that communities with higher species and functional diversity generally exhibit greater EMF (Fry et al., 2018).

### 4.2 Drivers of α and β diversity, functionality, and stability

Environmental change has profound impacts on long-term and extensive community assembly processes. These processes define appropriate habitats for species and shape their functional diversity (Brown and Milner, 2012). Our study revealed that environmental factors influence the species diversity (α-diversity) and community diversity (β-diversity) of the continuous successional sequences of alpine meadows and alpine steppes by 67% and 74%, respectively. This indicates that β-diversity is more influenced by environmental changes compared to α-diversity. Specifically, increased precipitation, soil water content, and soil nitrate-nitrogen levels were associated with higher plant species diversity. Conversely, higher altitudes, deteriorating topographic features like slope orientation and gradient, and reductions in available potassium were linked to decreased plant species diversity. Furthermore, altitude gains and higher soil conductivity enhanced community differentiation (β-diversity), while increases in precipitation, soil pH, and bulk density supported the consistency of plant communities. This pattern is likely due to altitude gains leading to diverse environmental conditions, prompting plant species to develop various adaptations and fostering community differentiation (Liu et al., 2020; Buckley et al., 2023). Additionally, higher soil conductivity may promote the aggregation of salt-tolerant plant communities, enhancing inter-community differentiation (Zhang et al., 2018a). Moreover, precipitation, soil pH, and bulk density improve plant community uniformity in this study.

Environmental factors significantly impact the functional diversity of communities (Portela et al., 2023). The study revealed that 67.30% of the variation in Functional Dispersion (Fdis) can be attributed to environmental factors, including mean annual precipitation (MAP), altitude, total nitrogen (TN), organic matter (OM), available potassium (AK), and electrical conductivity (EC). This implies that moisture, temperature, and soil nutrients play a crucial role in regulating the ecological niche differentiation of vegetation (Qi et al., 2015). Additionally, environmental variables explained 64.45% of the variance in Functional Richness (Fric), while Functional Divergence (Fdiv) and Evenness (Feve) explained 50.70% and 40.74%, respectively. Our findings show that the environment significantly influences the differentiation of vegetation ecological niches, followed by spatial utilization, ecological niche complementarity, and, to a lesser extent, resource use efficiency. Our study found that community resilience (ET) and resistance (RT) were significantly affected by environmental factors (*P* < 0.001). It was observed that environmental factors had a greater impact on ET (77.45% explained) than on RT (67.16% explained), indicating the crucial influence of environmental changes on ET. Subsequent analyses showed that higher mean annual temperature (MAT) and annual precipitation (MAP) increased the variability of community ET. Simultaneously, altitude was found to decrease the variability of community ET and RT.

### 4.3 Factors influencing the ecosystem multifunctionality of plant communities

Our research found a strong positive correlation between α-diversity and Fdis, Fric, Feve, RT, and EMF. This suggests higher species diversity enhances functional diversity, RT, and EMF. Conversely, greater community differences (β-diversity) showed a significant negative correlation with Fdis, Fdiv, ET, RT, and EMF, indicating that increased differences among communities can weaken functional diversity, ET, and RT, ultimately reducing EMF. Furthermore, functional diversity, ET, and RT affected EMF significantly and positively. Fdis had the highest impact (0.81***), followed by α-diversity (0.78***), RT (0.63***) and ET (0.59***) (**Supplementary Table 2**). Additionally, Fric, Feve and Fdiv were noted to impact EMF positively. The results suggest that high levels of niche differentiation within communities have the most significant positive impact on EMF, followed by species diversity. Our study also found that functional diversity reflects EMF more than species diversity under the degradation succession between AM and AS, supporting previous research findings (Zirbel et al., 2019). Moreover, the study found that Fdis had a stronger positive impact on RT than α-diversity. This suggests that functional diversity is a better indicator of community stability than species diversity during degradation succession. The results support the notion that increased diversity can protect against external threats, highlighting the importance of functional diversity over species diversity in ensuring community stability (Kraft et al., 2015). These findings enhance our understanding of the interactions among species diversity, functional diversity, community stability, and EMF throughout community degradation succession.

EMF describes an ecosystem’s capacity to provide functions such as productivity, nutrient cycling, and decomposition, assessing ecosystem health, and enhancing management and conservation efforts (Hölting et al., 2019; Van der Plas, 2019). Previous research has examined how species diversity, functional diversity, and climate independently influence changes in EMF (Jing et al., 2015; Steudel et al., 2016; Hertzog et al., 2019). However, the multifaceted nature of EMF drives the complexity of their underlying mechanisms, which are affected by multiple factors, making research more difficult (Giling et al., 2019). Consequently, it becomes increasingly imperative to elucidate the mechanisms underlying the multifunctionality of ecosystems. For our investigation, we considered various predictor variables such as habitat characteristics, species diversity, functional diversity, and community stability. Utilizing the random forest algorithm to identify the most important factors that influence changes in EMF. Based on the crucial factors identified and constructed, a mixed linear model was used to quantitatively assess their specific contributions to changes in EMF. The results showed that these factors explained 92.86% of the variations in EMF. Specifically, habitat factors accounted for 21.29% of the observed changes, while species diversity explained 32.95%, functional diversity 37.25%, and community stability 8.55% (**Figure 3E**). Significant linear correlations were found between Fdis, Fdiv, Fric, Feve, Patrick, and EMF. However, no other indicators showed any significant linear relationships. EMF is a complex phenomenon, so only a few single factors displayed significant linear correlations with it. Instead, composite factors such as climate, geography, and soil contribute more substantially to explaining changes in EMF.

In this study, we found that precipitation is a significant environmental differentiator between alpine meadows and alpine steppes in a continuous successional sequence. Precipitation was closely associated with EMF dynamics, as revealed by random forest analysis (**Figure 3A**). Meanwhile climate explaining 21.29% of the observed variation in EMF (**Figure 3E**). Notably, in our SEM, precipitation showed a strong positive correlation with EMF (0.543***) (**Figure 4**), suggesting that when precipitation is the dominant factor, changes in EMF within grassland ecosystems align with global dryland patterns—higher moisture availability enhances EMF in drylands globally (Maestre et al., 2012). Moreover, compared to temperature and nutrient changes, the impact of precipitation on dryland EMF was found to be more pronounced (Delgado-Baquerizo et al., 2017).

Our findings also highlighted that both functional diversity and species diversity significantly explained community EMF variation, with functional diversity (R^2^ = 64.90%, *P* < 0.01; **Figure 3C**) and species diversity contributing (R^2^ = 57.13%, *P* < 0.01; **Figure 3B**), respectively. These results align with previous studies showing that multiple dimensions of diversity—species, functional, and phylogenetic—are significantly correlated with EMF (De Bello et al., 2010; Zavaleta et al., 2010). Importantly, our research emphasizes that functional diversity has a greater influence on community EMF compared to species diversity (37.25% > 32.95%, **Figure 3E**). This is further supported by our SEM, where functional diversity (0.554***) exerted a stronger effect on EMF than species diversity (0.48***) (**Figure 4**). Our findings corroborate previous studies, which have emphasized that higher levels of species diversity are essential to maintain multiple ecosystem functions and that functional traits are more responsive to environmental changes, making them better predictors of EMF (Isbell et al., 2011; Pérez-Harguindeguy et al., 2013; Steudel et al., 2016; Zavaleta et al., 2010). These results align with findings from studies in Inner Mongolia, where drought significantly affected grassland vegetation, demonstrating that plant functional diversity, particularly traits such as plant height and leaf characteristics, explains EMF more effectively, while phylogenetic diversity contributes the least (Yan et al., 2020). Our study further supports previous findings that, under naturally assembled communities, functional diversity is a stronger driver of ecosystem multifunctionality compared to species diversity (Van der Plas, 2019). This can be attributed to the fact that functional diversity is directly linked to ecosystem functions and reflects the interactions between species and the environment, thereby best representing the influence of organisms on ecosystem processes (Steudel et al., 2016). For example, Gross found that skewness–kurtosis models incorporating trait abundance distributions outperformed species richness in predicting ecosystem multifunctionality, highlighting functional diversity as a major driver of ecosystem functions (Gross et al., 2017). Moreover, they observed that ecosystem multifunctionality reached its peak when the skewness of trait abundance distribution was zero. In summary, our findings indicate that in precipitation-driven vegetation ecosystems, functional diversity has a greater impact on EMF than species diversity. This underscores the importance of considering trait-based measures to better understand and predict ecosystem responses to environmental changes.

Furthermore, the study found a significant positive relationship between species diversity and functional diversity (0.577***) (**Figure 4**), supporting the notion that greater species diversity leads to a wider array of traits, enhancing functional trait diversity (Díaz and Cabido, 2001). Interestingly, our findings indicated that species and functional diversity had a positive yet insignificant effect on community stability. We speculate that the following processes might be involved: 1) Under favorable environmental conditions, species diversity and functional diversity might contribute insignificantly to stability due to high redundancy (Mori et al., 2013). 2) Conversely, under unfavorable environmental conditions—such as low soil nutrient levels, moisture, and temperature availability—the contribution of species diversity and functional diversity to stability may be limited (Jing et al., 2022; Pennekamp et al., 2018; Jing et al., 2022).

In addition, our study found that community stability explained 8.55% of the variance in grassland EMF (**Figure 3E**). The SEM further demonstrated that community stability had a significant positive effect on EMF (0.2*), specifically through community resistance (RT), which exhibited a notable positive effect (0.5*) on EMF (**Figure 4**). This finding was somewhat unexpected, revealing that community stability may play a more crucial role in maintaining grassland ecosystem multifunctionality than previously recognized. Previous studies have rarely explored the relationship between community stability and EMF. According to the biodiversity–stability hypothesis (Tilman and Downing, 1996), increased biodiversity is generally linked to enhanced ecosystem stability and functionality. We hypothesize that stable communities may more efficiently utilize resources, thereby minimizing niche overlap and enhancing overall ecosystem performance (Cardinale et al., 2012). Furthermore, community stability may help buffer against ecological disturbances, maintaining ecosystem function under varying conditions (Isbell et al., 2015). In response to environmental stressors such as drought or pest outbreaks, stable communities could rely on inherent resistance mechanisms to sustain ecological functions without significant impact. Interestingly, our findings diverge from previous research, which suggested that stability influences EMF indirectly through functional diversity (Glidden et al., 2023; Jia et al., 2023). In the specific context of alpine meadows and alpine steppes, which represent a continuous successional sequence, we observed that community stability directly affects EMF, challenging these earlier conclusions.

## 5 Conclusions

Our findings demonstrate that the successional transition from alpine meadows to alpine steppes is characterized by a marked reduction in species diversity, functionality, and stability, driven primarily by declining precipitation, soil nutrient depletion, and shifting soil acidity. These environmental perturbations orchestrate a complex restructuring of community composition and functional traits, affecting community resilience and resistance, and ultimately constraining ecosystem multifunctionality. Surprisingly, alongside the pivotal role of functional diversity and species richness, community stability emerges as a previously underappreciated but significant determinant of ecosystem multifunctionality.

Building on these insights, our results highlight the urgency of conserving not only species richness but also the functional attributes and stability of alpine grassland communities. This broader perspective will be essential for sustaining the integrated suite of ecosystem processes under intensifying climatic pressures. Future research should further elucidate the complex feedbacks among environmental drivers, community assembly, and multifunctionality, both within alpine systems and across other vulnerable biomes. Ultimately, these efforts will guide more effective management interventions that bolster ecological resilience and safeguard the biodiversity and productivity essential for sustaining ecosystem function under changing environmental conditions.

## Data Availability

The raw data supporting the conclusions of this article will be made available by the authors, without undue reservation.
