# Supplementary material for: Diversity, functionality, and stability: shaping ecosystem multifunctionality in the successional sequences of alpine meadows and alpine steppes on the Qinghai-Tibet Plateau
Source: Front Plant Sci. 2025 Mar 13;16:1436439. doi: 10.3389/fpls.2025.1436439 (PMC11966483; doi:10.3389/fpls.2025.1436439)
Supplement: Supplementary file 1 [file DataSheet1.docx]

Supplementary Material

**Diversity, Functionality, and Stability: Shaping Ecosystem Multifunctionality in the Successional Sequences of Alpine Meadows and Alpine Steppes on the Qinghai-Tibet Plateau**

Xin Jin, Abby Deng, Yuejun Fan, Kun Ma, Yangan Zhao, Yingcheng Wang, Kaifu Zheng, Xueli Zhou, Guangxin Lu^*^

*** Correspondence:** Guangxin Lu, E-mail: lugx74@qq.com

# Supplementary Figure

#
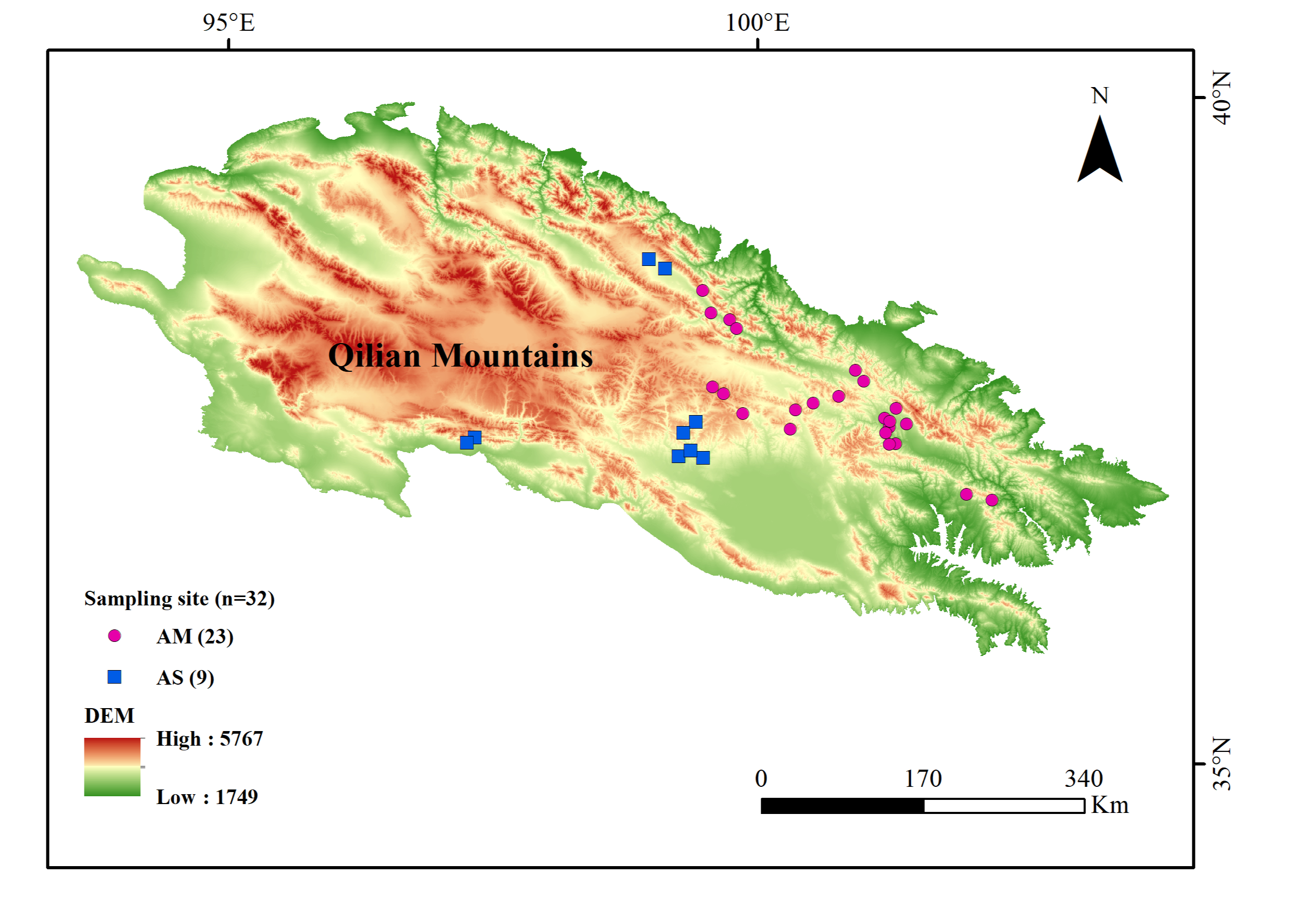


**Figure 1. Sampling site distribution map.**

**
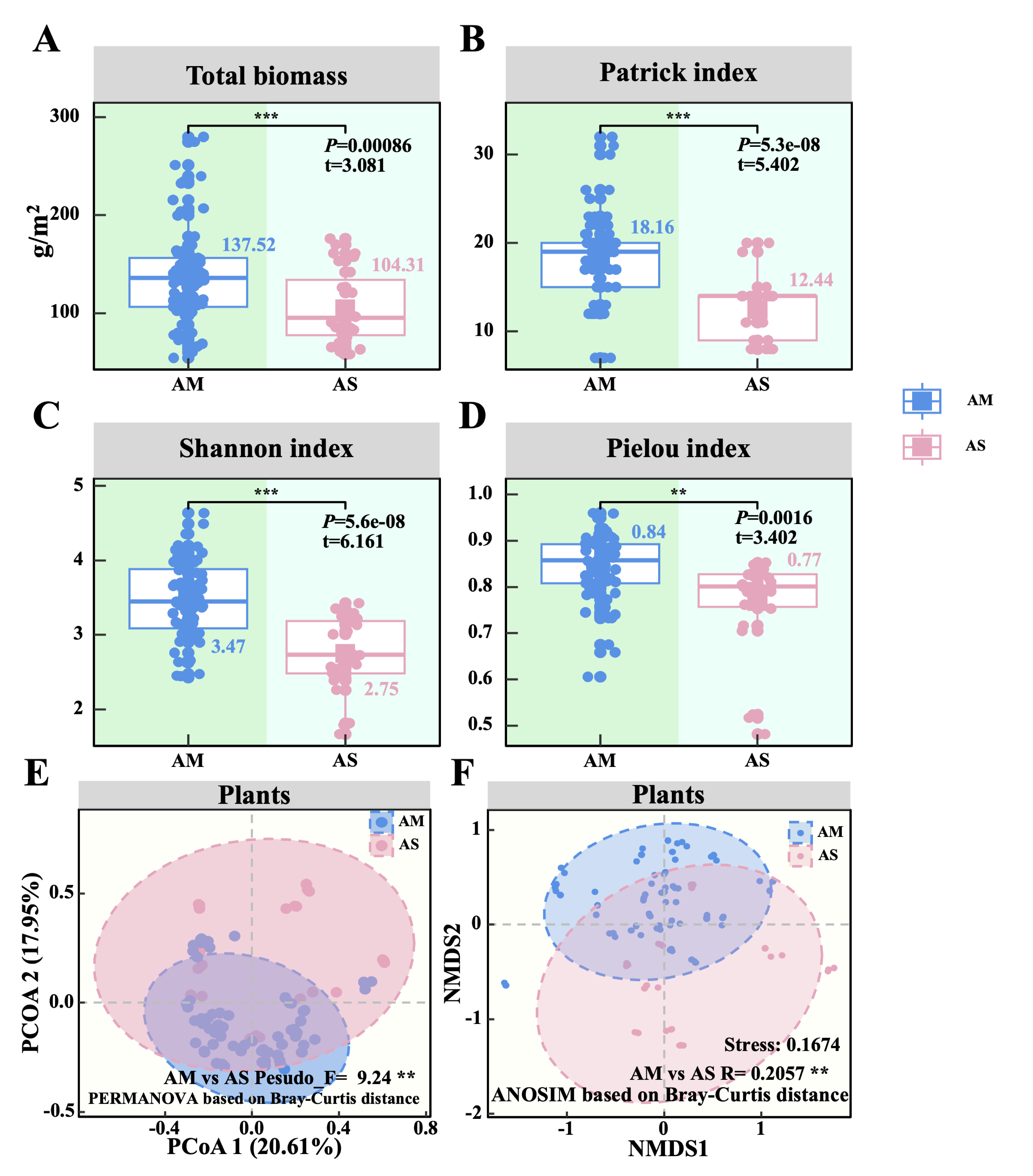
**

**Figure 2. Variations in α-diversity and β-diversity between alpine meadows (AM) and alpine steppes (AS).** (A) Total biomass; (B) Patrick index; (C) Pielou index; (D) Shannon index; (E) Principal Coordinate Analysis (PCoA); (F) Non-metric Multidimensional Scaling (NMDS). The values in figures (A)- (D) represent the means. Two treatments were compared—AM (n = 69) and AS (n = 27)—using an independent two-sample t-test (df = 94).


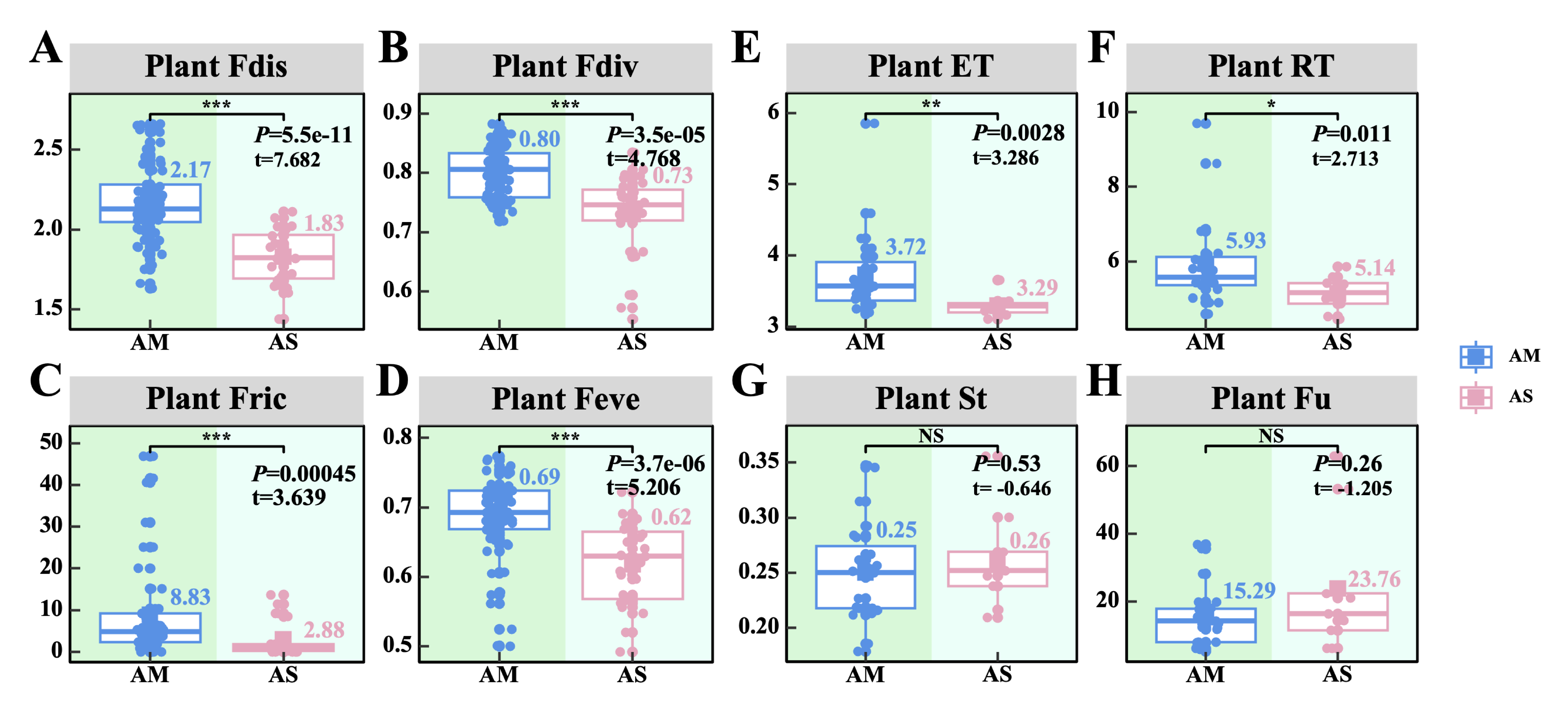


**Figure 3. Variations in functional diversity and community stability between alpine meadows (AM) and alpine steppes (AS).** (A) Plant functional dispersion (Fdis); (B) Plant functional divergence (Fdiv); (C) Plant functional richness (Fric); (D) Plant functional evenness (Feve); (E) Plant resilience (ET); (F) Plant resistance (RT); (G) Plant structural variability (St); (H) Plant functional variability (Fu). The values in panels (A)–(H) represent mean. Comparisons were made between two treatments, AM (n = 69) and AS (n = 27), using an independent two-sample t-test (df = 94).

#
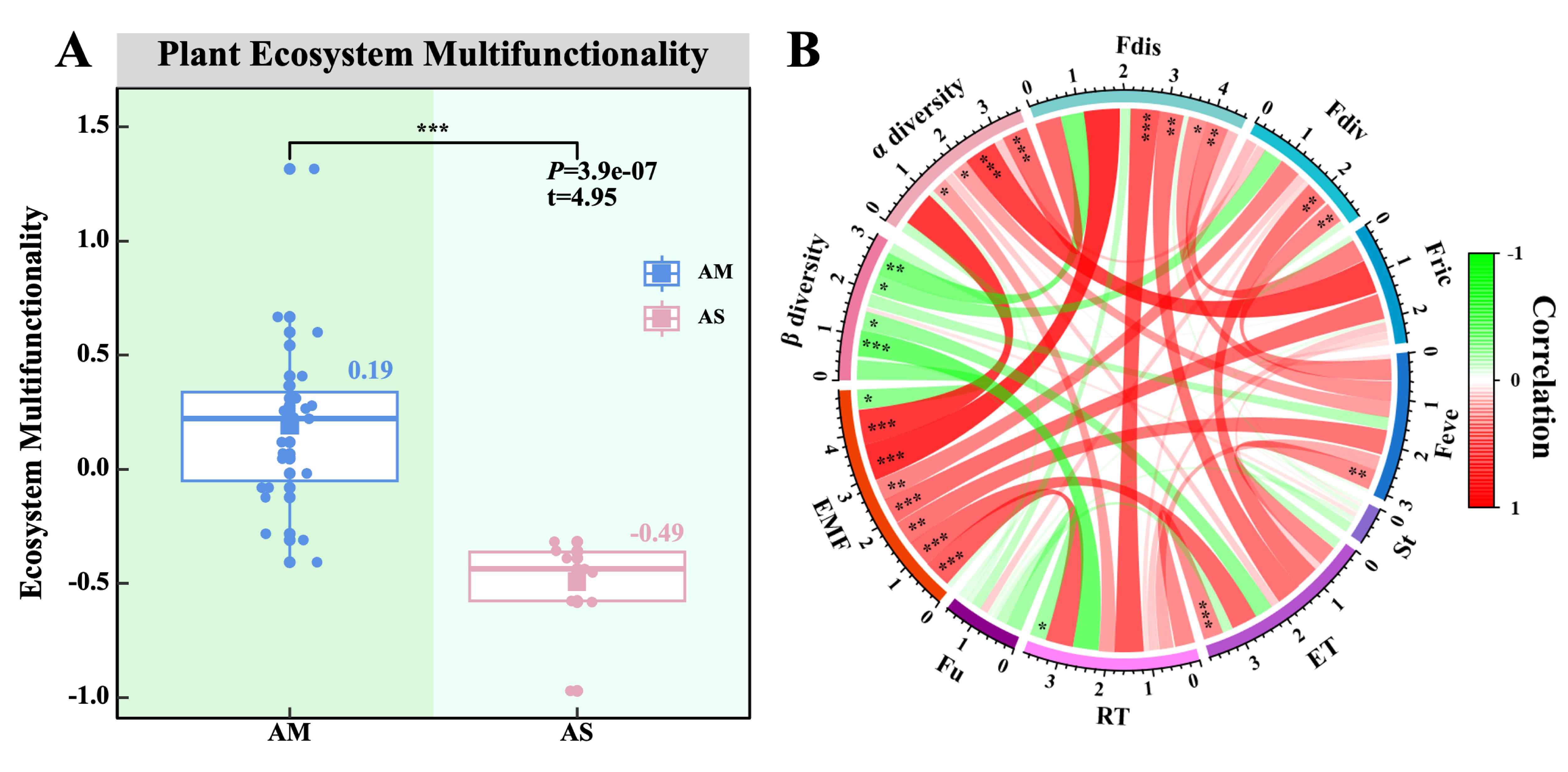


**Figure 4. Plant ecosystem multifunctionality (EMF) and its relationships with diversity and stability metrics in alpine meadows (AM) and alpine steppes (AS).** (A) Ecosystem multifunctionality in AM and AS. (B) Relationships between α-diversity, β-diversity, functional diversity, community stability, and EMF of plant communities along the degradation succession from alpine meadows (AM) to alpine steppes (AS).

In (A),the values represent the means, comparisons were made between two treatments, AM (n = 69) and AS (n = 27), using an independent two-sample t-test (df = 94). In (B), red lines indicate positive correlations, while green lines indicate negative correlations, visually distinguishing between the relationships. Heatmaps show Spearman's correlations between environmental factors, functional diversity, and community stability, with significant correlations marked by an asterisk (*). Asterisks indicate the level of significance (**P* < 0.05;***P* < 0.01;****P* < 0.001).

# Supplementary Tables

**Table S1. Summary of environmental factors in the study area; their units and ranges.**

| **Climatic variables** | **Range** |
| --- | --- |
| Annual mean temperature [°C] | -7.50 to -0.39 |
| Annual mean precipitation [mm] | 236.18 to 550.34 |

**Table S2.** **Relationships between α-diversity, β-diversity, functional diversity, stability, and ecosystem multifunctionality (EMF) of plant communities.** This table presents the detailed calculation results for **Figure 3B**, showing the relationships among diversity metrics, stability, and EMF along the degradation succession from alpine meadows (AM) to alpine steppes (AS).

| **Variant1** | **Variant2** | **Spearman Correlations** | **Significance** |
| --- | --- | --- | --- |
| EMF | β diversity | -0.43915 | * |
| EMF | α diversity | 0.77676 | *** |
| EMF | Fdis | 0.813783 | *** |
| EMF | Fdiv | 0.478006 | ** |
| EMF | Fric | 0.566716 | *** |
| EMF | Feve | 0.546921 | ** |
| EMF | ET | 0.585411 | *** |
| EMF | RT | 0.625 | *** |
| β diversity | Fdis | -0.52713 | ** |
| β diversity | Fdiv | -0.43658 | * |
| β diversity | ET | -0.41349 | * |
| β diversity | RT | -0.57735 | *** |
| α diversity | Fdis | 0.579912 | *** |
| α diversity | Fric | 0.745968 | *** |
| α diversity | Feve | 0.375 | * |
| α diversity | RT | 0.368035 | * |
| Fdis | Fric | 0.495968 | ** |
| Fdis | Feve | 0.42632 | * |
| Fdis | ET | 0.550587 | ** |
| Fdis | RT | 0.65176 | *** |
| Fdiv | Feve | 0.472507 | ** |
| Fdiv | ET | 0.540689 | ** |
| Feve | ET | 0.451613 | ** |
| ET | RT | 0.45198 | ** |
| RT | Fu | -0.36547 | * |

Asterisks indicate the level of significance (**P* < 0.05;***P* < 0.01;****P* < 0.001).
